# Boosting the Quantum Efficiency of Ionic Carbon Nitrides in Photocatalytic H_2_O_2_ Evolution via Controllable *n* → π* Electronic Transition Activation

**DOI:** 10.1002/adma.202412753

**Published:** 2024-10-17

**Authors:** Haijian Tong, Jokotadeola Odutola, Junsheng Song, Lu Peng, Nikolai Tkachenko, Markus Antonietti, Christian Mark Pelicano

**Affiliations:** ^1^ Department of Colloid Chemistry Max Planck Institute of Colloids and Interfaces 14476 Potsdam Germany; ^2^ Chemistry and Advanced Materials Faculty of Engineering and Natural Sciences Tampere University Tampere 33101 Finland

**Keywords:** defect sites, ionic carbon nitrides, oxamide, photocatalytic H_2_O_2_ evolution

## Abstract

Hydrogen peroxide (H_2_O_2_) is a crucial chemical used in numerous industrial applications, yet its manufacturing relies on the energy‐demanding anthraquinone process. Solar‐driven synthesis of H_2_O_2_ is gaining traction as a promising research area, providing a sustainable method for its production. Herein, a controllable activation of *n* → π* electronic transition is presented to boost the photocatalytic H_2_O_2_ evolution in ionic carbon nitrides. This enhancement is achieved through the simultaneous introduction of structural distortions and defect sites (─C ≡ N groups and N vacancies) into the KPHI framework. The optimal catalyst (*2%Ox‐*KPHI) reached an apparent quantum yield of 41% at 410 nm without the need for any cocatalysts, outperforming most previously reported carbon nitride‐based photocatalysts. Extensive experimental characterizations and theoretical calculations confirm that a corrugated configuration and the presence of defects significantly broaden the light absorption profile, improve carrier separation and migration, promote O_2_ adsorption, and lower the energy barriers for H_2_O_2_ desorption. Transient absorption spectroscopy indicates that the enhanced photocatalytic performance of *2%Ox*‐KPHI is largely attributed to the preferential migration of electrons at defect sites over extended timescales, following the diffusion of geminate carriers across the PHI sheets.

## Graphical abstract

A controllable activation of *n* → π* electronic transition is presented to boost the photocatalytic H_2_O_2_ evolution in ionic carbon nitrides through the simultaneous introduction of structural distortions and defect sites. The best catalyst (*2%Ox‐*KPHI) shows an apparent quantum yield of 41% at 410 nm without the need for any cocatalysts, outperforming most previously reported carbon nitride‐based photocatalysts.

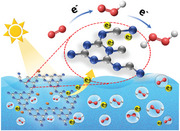

## 1 Introduction

As natural resources continue to deplete and energy demand increases, there is a need for large‐scale adoption of clean renewable energy sources.^[^ ^1^ ^]^ Hydrogen peroxide (H_2_O_2_) is widely valued for being an eco‐friendly, safe, and effective oxidizing agent. It is extensively used in medical applications, chemical synthesis, water treatment, and energy conversion technologies.^[^ ^2^, ^3^ ^]^ The global market value of H_2_O_2_ is estimated to be $3.5 billion in 2024 and will grow to $4.6 billion in 2028.^[^ ^4^ ^]^ Nowadays, industrial production of H_2_O_2_ primarily relies on anthraquinone method. However, this technique is complicated to manage and requires significant safety precautions. Artificial photosynthesis provides a sustainable and greener alternative route by harnessing sunlight to generate solar fuels and chemicals. This method utilizes photoexcited carriers to drive redox reactions preferably within an aqueous medium.^[^ ^5^, ^6^, ^7^, ^8^ ^]^

Among the numerous semiconductor photocatalysts developed to date, polymeric carbon nitride materials stand out owing to their adjustable chemical and catalytic properties, which can be fine‐tuned through strategies such as molecular modification, doping, and surface engineering.^[^ ^9^, ^10^, ^11^ ^]^ Specifically, ionic carbon nitrides like poly (heptazine imides) (PHI) have emerged as promising photocatalysts due to their highly crystalline structure and broader light absorption range.^[^ ^12^, ^13^, ^14^, ^15^ ^]^ In general, carbon nitrides can thermodynamically reduce O_2_ into H_2_O_2_ under suitable photocatalytic conditions via 2e^−^ O_2_ reduction reaction (ORR) route.^[^ ^16^, ^17^, ^18^, ^19^ ^]^ Based on this principle and prior research into the use of carbon nitrides for H_2_O_2_ production, Rogolino et al. initially examined protonated PHI (HPHI) and various metal PHIs (M = Fe^3+^, Ni^2+^, Co^2+,^ and Ru^3+^) through cation exchange with NaPHI as photocatalysts for ORR. NaPHI and HPHI achieved apparent quantum yields (AQYs) of 0.45% and 0.86% at an excitation wavelength of 410 nm.^[^ ^20^ ^]^ The authors confirmed that functionalizing PHI with transition metals is not favorable for H_2_O_2_ production as it promotes its decomposition. Hybridizing potassium PHI (KPHI) with adenine‐derived carbon material pushed its AQY to 1.1% at 410 nm.^[^ ^21^ ^]^ Although PHI materials have shown promise in photocatalytic H_2_O_2_ production, these recent achievements suggest that there is still considerable room to further refine and improve their light absorption properties.

In comparison to the heteroatom doping and forming heterojunction to extend the light absorption, inducing an *n* → π* electronic transition in carbon nitrides exemplifies a tactical dopant‐free and straightforward approach to enhance light absorption for H_2_O_2_ evolution. However, this method has not been extensively explored, due to the significant challenge posed by the fact that this electronic transition is not allowed in the perfectly symmetric and planar structure of carbon nitride.^[^ ^22^ ^]^ This is because the lone pair of electrons on N atoms are oriented orthogonally to the π ‐conjugation plane, preventing a photoexcitation to the conduction band.^[^ ^23^ ^]^ A viable method to enable *n* → π* electronic transition is by introducing structural distortions in carbon nitride. This alteration repositions the lone pair of electrons into a plane that permits transitions to π* orbitals. Several synthetic strategies to generate structural distortions have been reported including exfoliation and post‐synthesis thermal treatment of carbon nitrides.^[^ ^24^, ^25^, ^26^ ^]^ However, the impact of distorted heptazine units on the structural and photo(electro)chemical properties of PHIs remains poorly understood. Therefore, designing a controllable approach to modulate the *n* → π* electronic transition is essential for maintaining maximal structural order in PHIs. Moreover, employing defect engineering is a well‐established way to augment photocatalytic performance. The functionalization of carbon nitrides via introduction of surface defects including surface vacancies (C or N vacancies) and surface functional groups (─C ≡ N or ─NH_2_), can significantly enhance the selectivity and activity of photocatalytic reactions.^[^ ^27^, ^28^, ^29^, ^30^ ^]^

In this work, we demonstrate a route to enhance the photocatalytic H_2_O_2_ performance in ionic carbon nitrides by means of *n* → π* electronic transition activation. At the same time, the creation of structural distortions and defect sites (─C ≡ N group and N vacancies) within the KPHI framework led to significant improvement in light absorption, charge separation and migration, and accelerated oxygen reduction reaction (ORR) kinetics on the photocatalyst surface. Without any cocatalyst, the optimal catalyst (*2%Ox‐*KPHI) reached an AQY of 41% at 410 nm even with the moderately weak glycerin as the sacrificial agent, surpassing most other carbon nitride‐based photocatalysts. DFT calculations revealed that the build‐up of defect sites promotes the adsorption of O_2_ and the subsequent desorption of H_2_O_2_ on the surface of KPHI.

## 2 Results and Discussion

### 2.1 Morphological and Structural Characterization

**Figure 1:**
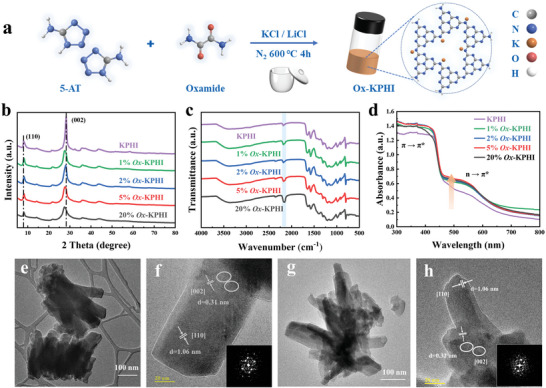
a) Illustration for the synthesis process of *x%Ox*‐KPHI. b) XRD patterns, c) FTIR spectra, and d) UV‐vis DRS spectra of KPHI and oxamide‐modified KPHI samples. HRTEM images of e,f) KPHI and g,h) *2%Ox‐*KPHI.

The synthetic process for preparing oxamide‐modified KPHI is shown in **Figure** **1**a. A specific amount of oxamide is combined with 2.5 g of 5‐aminotetrazole and 12.5 g of KCl/LiCl salt melt (mass ratio 0.55/0.45). This mixture was placed in a ball mill container and ground at a frequency of 25 Hz for 5 min. The resulting powder was then transferred into a crucible covered with a lid and annealed at 600 °C for 4 h under a flow of N_2_. The yellowish‐brown powders obtained are labeled as *x%Ox‐*KPHI, where x indicates the wt.% of oxamide added relative to 5‐aminotetrazole (Figure S1, Supporting Information). For comparison, pristine KPHI was prepared via the same procedure as above without the addition of oxamide (please see SI for detailed description). The powder X‐ray diffraction (XRD) patterns show two major distinct characteristic peaks (Figure 1b). The (110) and (002) crystal planes of KPHI are centered at 8.1 and 28.1°, respectively, which are attributed to the in‐plane long‐range order between tri‐s‐triazine units and periodic stacking of conjugated aromatic systems.^[^ ^21^ ^]^ Compared with KPHI, the (002) peak of the oxamide‐modified KPHI samples broadens and shifts toward lower angles, suggesting an expansion in interlayer distance. Thermogravimetric analysis suggests that oxamide might interfere with the polymerization process due to the gases released during its decomposition (Figure S2, Supporting Information). This disruption is evidenced by the reduced intensity of the XRD characteristic peaks, indicating that numerous defects were introduced into their structures and led to a lower degree of crystallinity.

Fourier transform infrared (FTIR) spectroscopy (Figure 1c) was used to analyze changes in the chemical structure of the prepared catalysts. The oxamide‐modified KPHI samples exhibit the same IR peaks as the original KPHI, demonstrating that their overall chemical structures remain unchanged. A notable peak at 801 cm^−1^ corresponds to the out‐of‐plane breathing vibration of the heptazine ring. The absorption bands at 987 and 1065 cm^−1^ are associated with the symmetric and asymmetric vibrations of NC₂ bonds of K─NC_2_ groups. The region from 1254 to 1500 cm^−1^ showcases stretching vibrational peaks assigned to the C─N and C═N bonds in the structure. Additionally, the N─H vibrational peaks found at 1575 and 1650 cm^−1^ arise from the protonated primary amine (H_2_NC) and secondary amine (HNC_2_) groups, respectively, indicating the presence of terminal and linking groups in the material. The broad vibrational peak in the region of 2900–3500 cm^−1^ is ascribed to ─NH_x_ and surface ─OH groups. A detailed examination of the asymmetric stretching vibration of the cyano group (─C ≡ N) at 2185 cm^−1^ shows that its intensity increases with higher oxamide content. This means that the ─NH_x_ groups in the KPHI structure are deprotonated into ─C ≡ N groups following the molten‐salt treatment of the precursor.^[^ ^31^ ^]^ Incorporating an optimal level of cyano defects is known to boost photocatalytic performance. Cyano groups can adjust the electronic structure and band gap of the material while also acting as trapping sites that anchor photogenerated charge carriers, thereby improving the overall efficiency.^[^ ^31^ ^]^ As shown in scanning electron (SEM) and transmission electron microscopy (TEM) images (Figure S3, Supporting Information), the addition of oxamide partially altered the rod‐like structure of KPHI. The nanorods appear more loosely packed and bent, possibly due to the release of gases during the pyrolysis of the starting materials. High‐resolution TEM elemental mapping images confirm the uniform distribution of K, C, N, and O elements across the material (Figure S4, Supporting Information).

As more oxamide is added, there is a noticeable rise in the C content and a corresponding decrease in N content in the samples, as shown in Table S1 (Supporting Information). More specifically, introducing an appropriate amount of oxamide enhances the C/N ratio, which denotes that N vacancies are formed during the thermal condensation process. However, the addition of 20 wt.% oxamide lowers the C/N ratio, implying that too much oxamide could decrease the polymerization extent of KPHI, as supported by the XRD results. Closer inspection using high‐resolution TEM images (Figure 1e–g) reveals that the introduction of oxamide induces partial distortion or corrugation in the nanorod structure, deviating from the original straight rod‐like form typically observed in KPHI. Both KPHI and a representative oxamide‐modified KPHI sample (*2%Ox*‐KPHI) exhibit characteristic lattice spacings of 1.06 and 0.31 nm, corresponding to the (110) and (001) crystal planes. All the oxamide‐modified KPHI samples demonstrate larger specific surface areas and pore sizes compared to the pristine KPHI, (44.5 m^2^g^−1^, Figure S5 and Table S2, Supporting Information). Pore size distribution analysis indicates a substantial increase in pore volume following oxamide modification, which could enhance O_2_ adsorption and availability of active sites for photocatalytic H_2_O_2_ production. Nevertheless, when the oxamide content is raised to 20 wt.%, a reduction in both specific surface area and pore volume is observed. The removal of water during the transformation of oxamide to dicyanamide may have interfered with NH_3_ release during the polymerization process, compromising the structural integrity of PHI. These results correlate well with the structural looseness of its rod‐like crystals observed in the microscopy analysis.

To elucidate the differences in chemical structure and composition among the samples, X‐ray photoelectron spectroscopy (XPS) was performed. Based on the XPS survey spectrum (Figure S6a, Supporting Information), all the synthesized catalysts contain C, N, O, and K elements, confirming that no elemental impurities are present. The high‐resolution C1s spectra can be deconvoluted into three distinct peaks at 284.8, 286.4, and 288.1 eV, which are assigned to carbon impurities (C─C), aminated carbons on the edge of aromatic units (C─NH_x_) and sp^2^ hybridized carbon (N─C═N) in the heptazine ring structure (Figure S6b, Supporting Information).^[^ ^32^ ^]^ It can be seen that the *2%Ox*‐KPHI sample shows a more pronounced C─NH_x_ peak. Considering that the binding energy of the ─C≡N group overlaps with that of the C─NH_x_ group, this could be linked to the higher density of ─C≡N group observed in the FTIR analysis. The N1s region can be fitted into four distinct peaks: the peaks at 398.5 and 399.4 eV are attributed to bi‐coordinated N (C─N═C, N_2_C) and tri‐coordinated N in the heptazine ring structure (N─C_3_, N_3_C), respectively.^[^ ^12^ ^]^ The peak detected at 400.7 eV corresponds to the N atoms present in the terminal ─NH_x_ or ─C≡N groups. The stronger signal at this binding energy for *2wt.%Ox*‐KPHI indicates an increase in ─C≡N groups as a result of oxamide modification (Figure S6c, Supporting Information). Moreover, increasing the oxamide content reduced the ratio between N_2_C and N_3_C, indicating the formation of N vacancies (Table S3, Supporting Information). UV‐vis diffuse reflectance spectroscopy (DRS) was conducted to evaluate how lattice distortion and alterations in the molecular structure affect the optical properties of the samples (Figure 1d). Compared to pristine KPHI, the oxamide‐modified KPHI samples showed noticeably stronger absorption bands originating from π → π* electronic transitions, and new absorption bands emerged due to *n* → π* transitions involving lone pair of electrons at the defect sites.^[^ ^23^ ^]^ This *n* → π* electronic transition extends the absorption edge from 455 nm for KPHI to ≈650 nm for *x%Ox*‐KPHI samples, narrowing the intrinsic band gap to ≈ 1.9 eV. To further explore this feature, room‐temperature electron paramagnetic resonance (EPR) spectroscopy was performed (Figure S7, Supporting Information). Both KPHI and *2%Ox*‐KPHI show a primary Lorentzian line with a g value of 2.003, typically attributed to the lone pair electrons on the sp^2^‐N atoms within the π‐conjugated CN aromatic rings.^[^ ^33^ ^]^ The more intense EPR signal observed for *2%Ox*‐KPHI implies an elevated concentration of unpaired electrons within an expanded π‐conjugated system.^[^ ^34^, ^35^ ^]^ Clearly, these results establish that lattice distortion and precise defect concentration adjustment can activate the *n* → π* electronic transition, enhance visible light absorption and increase electron density.

### 2.2 Photocatalytic Performance

**Figure 2:**
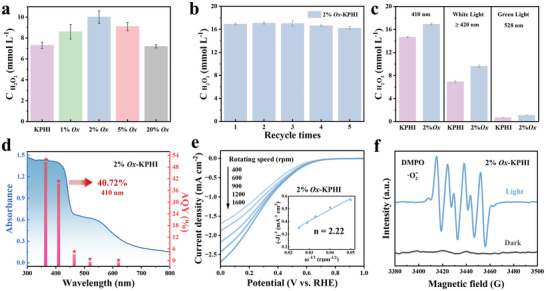
a) Influence of oxamide loading on the H_2_O_2_ evolution performance of KPHI. Reaction conditions: photocatalyst, 5 mg; solvent, 2 mL of 3.5% w/w glycerin bubbled with O_2_ for 1 min before the reaction; light source, violet LED (*λ* = 410 nm). b) Recyclability tests for *2%Ox*‐KPHI in H_2_O_2_ production. c) Photocatalytic H_2_O_2_ evolution activities of KPHI and *2%Ox*‐KPHI using different excitation wavelengths. Reaction conditions for b) and c): photocatalyst, 5 mg; solvent, 2 mL of 10% w/w glycerin bubbled with O_2_ for 1 min before the reaction; light source, violet LED (*λ* = 410 nm). d) UV‐vis spectrum and AQY values for *2%Ox*‐KPHI as a function of the irradiation wavelength. e) LSV curves of *2%Ox*‐KPHI tested by RDE (Inset: fitted K–L plot). f) EPR spectra of DMPO‐⋅O_2_^−^ over KPHI and *2%Ox*‐KPHI.

The photocatalytic H_2_O_2_ production activity of the as‐prepared samples was evaluated under a 410 nm LED irradiation without using any cocatalyst (**Figure** **2**a). Photocatalytic experiments are performed with the catalysts (2.5 mg mL^−1^) dispersed in O_2_‐saturated aqueous glycerin solution (3.5% w/w). Glycerin is an attractive alternative to conventional trialkyl amine sacrificial electron donors owing to its expected increased production from the biofuel industry. It undergoes a 2e⁻ oxidation process, converting into dihydroxyacetone or glyceraldehyde while simultaneously transferring electrons to the photocatalyst.^[^ ^20^ ^]^ No H_2_O_2_ is detected in the dark or without catalysts, emphasizing that photocatalysis is crucial for the process (Figure S8a, Supporting Information). Among the samples, *2%Ox*‐KPHI exhibited the highest activity (10 mM h^−1^) after 1 h irradiation, which is ≈40% higher than pure KPHI (7 mM h^−1^). However, raising the oxamide content beyond 2 wt.% resulted in a gradual drop in performance, attributed to decreased sheet sizes which enhance geminate recombination. These results highlight the pivotal role of oxamide modification in modulating the structure of KPHI with enhanced *n* → π* electronic transition, thereby boosting the overall catalytic efficiency. Adjusting the glycerin concentration to 10% w/w improved the activity, reaching 16.9 mM h^−1^ (Figure S8b, Supporting Information). The decline in H_2_O_2_ production after 60 min of irradiation might be ascribed to its decomposition, likely triggered by the higher temperature from prolonged light exposure (Figure S8c, Supporting Information). Besides its remarkable photocatalytic efficiency, *2%Ox*‐KPHI demonstrates excellent durability and reusability. As illustrated in Figure 2b, the H_2_O_2_ concentration shows only a minimal decrease after five consecutive reaction cycles, which could be associated to the loss of catalyst during each cycle of washing and recycling. Following recyclability tests, the color of the *2%Ox*‐KPHI transformed from yellowish‐brown to off‐white powder that signifies an alteration in the catalyst's structure or chemical composition. Another striking change is observed in the FTIR spectrum of the reused *2%Ox*‐KPHI catalyst: the disappearance of K─NC_2_ groups and emergence of new bands at 3250 and 2800 cm^−1^, associated with ─NH_x_ and ─CH group, respectively (Figure S9a, Supporting Information). Figure S9b (Supporting Information) shows a shift of the (002) diffraction peak toward lower angles indicative of in‐planar packing distance expansion.

These changes infer that protons from the oxidation of glycerin may have coordinated with the anionic sites on the photocatalyst surface, consistent with our previous findings.^[^ ^21^ ^]^ To examine the correlation between light absorption and the photocatalytic efficiency of the *2%Ox*‐KPHI catalyst compared to KPHI, we carried out wavelength‐dependent H_2_O_2_ evolution experiments (Figure 2c). Interestingly, the *2%Ox*‐KPHI catalyst exhibited a 40% increase in activity under white light (λ > 420 nm) and a 55% increase under green LED illumination (λ = 528 nm) relative to KPHI. This superior performance of *2%Ox*‐KPHI at longer wavelengths strongly suggests that the oxamide modification effectively enhances the light absorption capability of KPHI. The apparent quantum yield (AQY) is estimated to be 50% and 41% at 365 and 410 nm excitation wavelength, respectively (higher than KPHI: AQY_410 nm_ = 32%) Figure 2d). These values show that *2%Ox*‐KPHI catalyst is far superior to most reported carbon nitride‐based photocatalysts for H_2_O_2_ production (Table S4, Supporting Information).

It is well‐known that O₂ can receive an e⁻ to form ∙O_2_⁻, which then reacts with an additional e⁻ to produce H₂O₂ through a sequential two‐step, one‐electron O_2_ reduction reaction (ORR). The average e⁻ number (n) involved in the ORR is a vital parameter for analyzing the O₂ reduction pathway using electrochemical rotating disk electrode (RDE) measurements. We evaluated the electrocatalytic ORR activities of *2%Ox*‐KPHI and KPHI by linear sweep voltammetry in O_2_‐saturated 0.5 m Na_2_SO_4_ aqueous solution with different rotating speeds (Figure 2e; Figure S10a, Supporting Information). Within similar potential window, *2%Ox*‐KPHI exhibits higher cathodic current densities than KPHI, reflecting faster ORR kinetics on its surface. This observation is further validated by rotating ring‐disk electrode (RRDE) tests (Figure S10b, Supporting Information). *2%Ox*‐KPHI shows higher disk and ring currents relative to KPHI, indicating a faster ORR rate. The calculated electron transfer number from the slopes of the linearly fitted Koutecky‐Levich plots are determined to be 2.81 and 2.22 for KPHI and *2%Ox*‐KPHI, respectively. These values confirm that reduction of O_2_ to H_2_O_2_ predominantly follows a 2e⁻ pathway, rather than the 4e⁻ route associated with H_2_O oxidation. Compared with KPHI, it is evident that the electron transfer number of *2%Ox*‐KPHI is closer to 2 and its H_2_O_2_ selectivity is higher under identical conditions (Figure S11, Supporting Information).

To better understand the photocatalytic H_2_O_2_ production mechanism, we conducted free radical trapping tests using AgNO_3_, tert‐butyl alcohol (TBA), and Na_2_S_2_O_3_ (Figure S12, Supporting Information). These compounds were employed as scavengers for electrons (e⁻), superoxide radicals (∙O_2_⁻), and hydroxyl radicals (•OH), respectively. Using N_2_ to purge the reaction mixture instead of O_2_ results in almost no detection of H_2_O_2_, indicating that O_2_ is essential for the reaction to proceed. Similarly, the addition of AgNO_3_ significantly inhibits the H_2_O_2_ production rate which highlights the critical role of e⁻ in generating H_2_O_2_. The addition of TBA cuts the H_2_O_2_ yield by half, denoting that •OH radicals play a partial role in H_2_O_2_ formation, which is verified by EPR measurement under light irradiation in Figure S13 (Supporting Information). As shown in Figure S12 (Supporting Information), detectable amounts of H_2_O_2_ can still be produced even without glycerin, suggesting that photoexcited holes can drive water oxidation and form •OH radicals. Simultaneously, this reaction releases protons from H_2_O molecules, which can further promote the hydrogenation process during the 2e⁻ ORR.^[^ ^18^ ^]^ When Na_2_S_2_O_3_ is introduced, H_2_O_2_ evolution drops to nearly zero, implying that •O_2_⁻ radicals are necessary intermediates in the H_2_O_2_ generation process. These results clearly show that H_2_O_2_ was formed through two consecutive 1e^–^ ORRs in the irradiated *2%Ox*‐KPHI catalyst system. We additionally validated the generation of •O_2_⁻ radicals through EPR measurements. As illustrated in Figure 2f, both KPHI and *2%Ox*‐KPHI catalysts show no signal in the dark. However, after being exposed to 410 nm LED light for 5 min, a response from the •O_2_⁻ radicals becomes visible. Notably, *2%Ox*‐KPHI shows a higher response intensity which verifies that the ─C ≡ N groups can effectively boost the conversion of O₂ to •O_2_⁻, thereby augmenting the production of H_2_O_2._

### 2.3 Optical and Photo(Electro)Chemical Characterization

**Figure 3:**
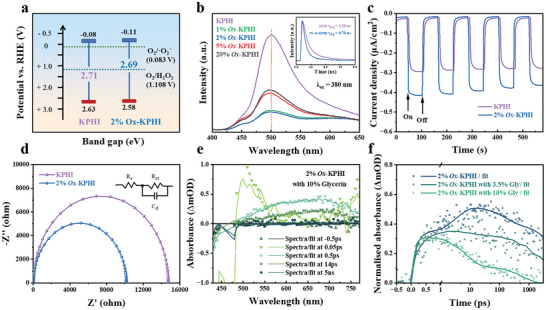
a) Experimentally determined band structures of KPHI and *2%Ox*‐KPHI. b) Room temperature steady‐state PL emission spectra of KPHI and *2%Ox*‐KPHI with an excitation wavelength of λ = 380 nm (inset: solid‐state time‐resolved PL decay of KPHI and *2%Ox*‐KPHI). c) Transient photocurrent (λ = 410 nm) for KPHI and *2%Ox*‐KPHI in 0.2 m Na_2_SO_4_ aqueous solution. d) Electrochemical impedance spectroscopy (EIS) Nyquist plots. e) Femtosecond transient absorption spectroscopy (fs‐TAS) absorbance spectra at different delay times for *2%Ox*‐KPHI in 10% Glycerin. (410 nm excitation, 0.03 mJ cm^−2^) f) fs‐TAS decays at 675 nm monitoring wavelength for *2%Ox*‐KPHI in different concentrations of Glycerin. (410 nm excitation, 0.03 mJ cm^−2^).

To clarify the increased photocatalytic performance occurring over *2%Ox*‐KPHI, optical and photo(electro)chemical measurements were conducted. The band structures of photocatalysts are critical indicators of their ability to facilitate redox reactions and their efficiency in separating charges. Therefore, the optical properties and band structure of the *2%Ox*‐KPHI catalyst were first analyzed. As discussed earlier, the introduction of oxamide extended the light absorption of KPHI by activating *n* → π* electronic transition and slightly narrowing its bandgap (2.71 eV for KPHI and 2.69 eV for *2%Ox*‐KPHI). Based on Mott‐Schottky analysis, the flat‐band potential (*E*_fb_) shifted cathodically which increases the thermodynamic force for O_2_ reduction (**Figure** **3**a).^[^ ^36^ ^]^

Next, steady‐state photoluminescence (PL), time‐resolved PL, transient photocurrent response, and electrochemical impedance spectroscopy (EIS) were performed to explore the charge separation and transfer dynamics within the *2%Ox*‐KPHI catalyst. When excited at a wavelength of 380 nm, pure KPHI displays a prominent PL emission peak at 500 nm, attributed to the radiative recombination of photoexcited charge carriers (Figure 3b). The intensity of this emission decreases progressively with the addition of oxamide. Presumably, this effect is due to the increased cyano (─C ≡ N) defects which can act as electron sinks and thus suppress radiative recombination through the *n* → π* transitions. However, once the oxamide content surpasses 2 wt.%, the PL signal intensifies again, indicating increased radiative recombination. This effect could be due to smaller sheet sizes with excess oxamide precursor which promotes increased geminate carrier recombination, because of shorter carrier diffusion lengths. Therefore, the charge carriers recombine before they have time to be captured at the ─C ≡ N groups. This finding agrees well with the photocatalytic activity measurements.

As shown in the inset of Figure 3b, the solid‐state time‐resolved fluorescence decay spectra reveal that *2%Ox*‐KPHI (0.78 ns) has a shorter average carrier lifetime than KPHI (1.36 ns). The decrease in the average charge lifetime implies suppression of the normal radiative recombination pathway due to increased electron confinement at the ─C ≡ N groups. In addition, the steady‐state photoluminescence at 410 nm excitation was also studied for KPHI and our representative oxamide sample, *2%Ox*‐KPHI with different glycerin concentrations (Figure S14a, Supporting Information). A drop in the PL intensity is also observed for the *2%Ox*‐KPHI compared to the KPHI, due to the suppression of radiative recombination from carrier capture at the ─C ≡ N groups. With increasing glycerin concentrations, an increase in the PL intensity is observed due to increased viscosity which suppresses the diffusion of oxygen in solution, minimizing electron scavenging and allowing increased radiative recombination. Consequently, the transient photocurrent response of *2%Ox*‐KPHI reveals improved availability of photogenerated carriers and increased current density in comparison to pristine KPHI (Figure 3c). Electrochemical impedance spectroscopy (EIS) measurements were carried out to further probe the charge transfer dynamics (Figure 3d). The smaller semicircle diameter for *2%Ox*‐KPHI indicates lower electric resistance, which is conducive to the charge transfer process.

In addition, femtosecond transient absorption spectroscopy (fs‐TAS) measurements were conducted on *2%Ox*‐KPHI suspensions, using 410 nm excitation across varying glycerin concentrations, to gain a deeper understanding of the early timescale dynamics (fs‐ns) that occur immediately after photon absorption. In the fs‐TAS differential spectra (Figure 3e), following the rapid thermalization of carriers, a strong negative signal was observed at <510 nm, which can be attributed to the ground state bleaching (GSB) of *2%Ox*‐KPHI and a broad positive signal at > 510 nm which can be attributed to the excited state absorption (ESA). As the time delay increased to up to 5 ns, the ESA developed further, revealing a prominent broad band centered at 675 nm. Based on similar studies involving ionic PHI suspensions,^[^ ^37^, ^38^ ^]^ this peak at 675 nm is attributed to the photogenerated excitons from the PHI nanosheets.

According to the fs‐TAS decays (Figure 3f), the ESA at 675 nm decays more rapidly as the amount of glycerin added increases. This accelerated decay within the first few 100 ps of the fs‐TAS response has also been reported in other ionic PHI systems when different hole scavengers, such as methanol and 4‐methylbenzyl alcohol, are introduced.^[^ ^37^, ^38^ ^]^ This faster decay is due to the generation of anionic species that quench excitons involved in geminate recombination along the sheet's direction.^[^ ^39^ ^]^ Therefore, the addition of glycerin suppresses geminate recombination on sub‐ns timescales by scavenging holes, which increases the availability of electrons for photocatalysis over longer timescales. The excitonic dynamics observed in the *2%Ox*‐KPHI suspensions are similar to those in KPHI suspensions, as shown by the fs‐TAS results in Figure S14b–f and Table S5 (Supporting Information). This similarity supports the idea that the superior photocatalytic performance of the *2%Ox*‐KPHI, compared to KPHI, is primarily due to the preferential migration of electrons at defect sites over longer timescales, after geminate carriers have migrated across the PHI sheets. Additionally, the fs‐TAS decays of KPHI (Figure S15, Supporting Information) show a similar pattern with different concentrations of glycerin, further confirming the quenching effect.

**Figure 4:**
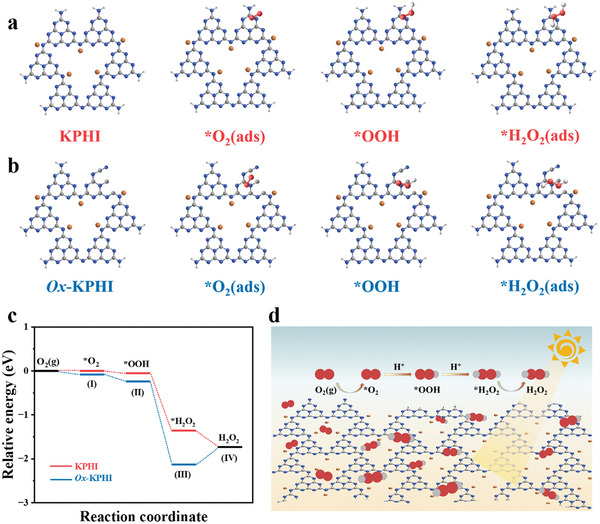
Optimized modeling for the reduction of O_2_ into H_2_O_2_ by an indirect two‐step single‐electron ORR process on a) KPHI and b) *2%Ox*‐KPHI. c) Relative energy diagrams for photocatalytic H_2_O_2_ over KPHI and *2%Ox*‐KPHI. d) Schematic diagram mechanism of H_2_O_2_ production over *2%Ox*‐KPHI through photocatalytic ORR.

To gain more insight into the key role of the defect sites (─C ≡ N group and N vacancies) in photocatalytic H_2_O_2_ evolution on both pristine KPHI and *2%Ox*‐KPHI catalysts, density functional theory (DFT) calculations were performed. This process undergoes the sequential transformation of *O_2_ (I) to *HOO (II), then to *H_2_O_2_ (III), and finally dissociation (IV) to reset the catalytic site for the next cycle.^[^ ^40^ ^]^ The optimized models for KPHI and *2%Ox*‐KPHI, shown in **Figure** **4**a,b, reveal that the active sites for O_2_ conversion to H_2_O_2_ are predominantly located on the N‐atoms and ─C ≡ N groups. The energy profiles for the intermediates on both catalysts are illustrated in Figure 4c. The initial energy of *O_2_ formation on KPHI is nearly zero (0.001 eV), suggesting a weak interaction between O_2_ and KPHI. This thermodynamic challenge in the first reaction step could impede the subsequent steps. In contrast, the formation energies of *O_2_ and *OOH on *2%Ox*‐KPHI are negative (−0.1 eV from I to II and −0.15 eV from II to III), indicating a stronger tendency for O_2_ to be adsorbed and converted on the surface of *2%Ox*‐KPHI.

The maximum energy released during *H_2_O_2_ production on *2%Ox*‐KPHI is −2.13 eV, significantly larger than the −1.36 eV observed on KPHI, underscoring the superior potential of *2%Ox*‐KPHI for H_2_O_2_ production. Although a small energy input of 0.27 eV is required to desorb H_2_O_2_ from the surface of *2%Ox*‐KPHI (steps III to IV), this barrier is likely overcome by the energy released in the earlier steps, enabling efficient H_2_O_2_ release. Figure S16 (Supporting Information) shows the effect of structural distortion on the PHI framework, causing a shift in the distances between the central N atoms in each heptazine unit, increasing from 7.4 to 7.6 Å for KPHI and 2%Ox‐KPHI, respectively. This distortion leads to an uneven distribution of local electron density, creating an electron‐rich environment around the N atoms in the isolated ─C ≡ N groups, which likely serve as active sites for the subsequent ORR reaction. Figure 4d shows the proposed mechanism for photocatalytic ORR leading to H_2_O_2_ production on *2%Ox*‐KPHI. The introduction of oxamide causes structural distortions inducing *n* → π* electronic transitions, broadening the light absorption range, and increasing the amount of ─C ≡ N group and N vacancies in the *2%Ox*‐KPHI structure. This results in greater photon absorption and generation of more charge carriers. As a result, these electrons are rapidly captured by the ─C ≡ N group and N vacancies, significantly enhancing electrons‐hole separation and thereby improving the overall solar‐to‐H_2_O_2_ conversion efficiency.

## 3 Conclusion

In summary, we developed a facile method to improve the solar‐driven H_2_O_2_ performance of ionic carbon nitrides by inducing *n* → π* electronic transition. Incorporating oxamide during polymerization distorted the KPHI crystals and generated structural defects such as ─C ≡ N group and N vacancies. At the same time, the buckled layered structure of oxamide‐modified KPHI showed extended light absorption to the visible range. The best‐performing catalyst (*2%Ox*‐KPHI) reached an apparent quantum yield of 41% at 410 nm without requiring any cocatalysts, surpassing the performance of most carbon nitride‐based photocatalysts reported to date. Based on optical and photoelectrochemical measurements, these structural alterations facilitated more efficient charge transport via preferential migration of electrons to the cyano defects and suppressing charge recombination. Electrochemical characterizations proved the faster ORR kinetics occurring over the surface of the oxamide‐modified photocatalyst. DFT calculations also revealed that the defect sites promoted O_2_ adsorption and H_2_O_2_ desorption, thereby augmenting the overall solar‐to‐H_2_O_2_ efficiency. Our findings highlight the potential of unlocking *n* → π* electronic transition, enabling the rational design high‐performance ionic carbon nitrides for photocatalytic H_2_O_2_ production.

## 4 Experimental Section

### Chemicals

All chemicals and solvents used were purchased from different chemical suppliers (Thermo Scientific, Merck, Sigma‐Aldrich) in high purity grade and were used as received. 5‐Amino‐1H‐tetrazole monohydrate was dried in a vacuum oven before use.

### Catalyst Preparation: Synthesis of KPHI

Potassium poly(heptazine imide) (KPHI) was synthesized following the methodology outlined in our prior publication.^[^ ^21^ ^]^ The procedure involved taking 2.5 g of dried 5‐Amino‐1H‐tetrazole monohydrate and 12.5 g of a KCl/LiCl eutectic mixture (in a 0.55/0.45 ratio) and placing them in a steel ball mill vessel. This mixture was then ground at an operational frequency of 25 Hz for 5 min. The resulting white powder was transferred to a porcelain crucible with a lid and heated in a furnace. The temperature of the furnace was gradually increased to 600 °C at a rate of 2.3 °C per min under a continuous flow of N_2_ gas (4 L min^−1^) and held at this temperature for 4 h. After the heating process, the furnace was allowed to cool down naturally to room temperature. The product was then transferred from the crucible into a beaker with deionized H_2_O and stirred at room temperature overnight. Following this, the mixture was vacuum filtered, washed extensively with water through centrifugation, and dried in a vacuum oven at 60 °C overnight.

### Catalyst Preparation: Synthesis of *x%Ox*‐KPHI

The synthesis of *x%Ox*‐KPHI followed a similar procedure to KPHI, with a minor modification in the salt template. Specifically, 1–20 wt.% (relative to the weight of 5‐Amino‐1H‐tetrazole) of oxamide was added to the KCl‐LiCl eutectic salt mixture to prepare Ox‐KPHI with varying oxamide content. The weight ratio of oxamide to 5‐Amino‐1H‐tetrazole was maintained between 1 and 20, and the subsequent steps were identical to those for KPHI. The resulting catalysts were designated as *x%Ox* ‐KPHI, where x represents 1, 2, 5, and 20, respectively.

The details of the characterization can be found in the Supporting Information.

## Conflict of Interest

The authors declare no conflict of interest.

## Supporting information



Supporting Information

## Data Availability

The data that support the findings of this study are available from the corresponding author upon reasonable request.
