# Supplementary material for: Boosting the Quantum Efficiency of Ionic Carbon Nitrides in Photocatalytic H2O2 Evolution via Controllable n → π* Electronic Transition Activation
Source: Adv Mater. 2024 Oct 17;36(49):2412753. doi: 10.1002/adma.202412753 (PMC11619226; doi:10.1002/adma.202412753)
Supplement: Supplementary file 1 — Supporting Information [file ADMA-36-2412753-s001.docx]

Supporting Information

Boosting the Quantum Efficiency of Ionic Carbon Nitrides in Photocatalytic H_2_O_2_ Evolution via Controllable $n\to\pi^{*}$ Electronic Transition Activation

*Haijian Tong ^1^, Jokotadeola Odutola ^2^, Junsheng Song^1^, Lu Peng,^1^ Nikolai Tkachenko ^2^, Markus Antonietti^1^, and Christian Mark Pelicano*^, 1^*

^1^Department of Colloid Chemistry, Max Planck Institute of Colloids and Interfaces, Potsdam 14476, Germany

^2^ Chemistry and Advanced Materials, Faculty of Engineering and Natural Sciences, Tampere University, 33101 Tampere, Finland

Corresponding author: christianmark.pelicano@mpikg.mpg.de

Experimental Section

1. General Characterizations

X-ray powder diffraction (XRD) patterns were measured using a Rigaku Smart Lab (Japan, Cu K, 0.154 nm) at a generator voltage of 40 kV and a generator current of 50 mA, with a scanning speed of 2°/min from 5° to 80°. X-ray photoelectron spectroscopy (XPS) spectra were performed via Thermo Fisher Scientific ESCALAB 250Xi. Fourier transform infrared (FTIR) spectroscopy measurements were conducted using a Thermo Scientific Nicolet iD5 spectrometer with an attenuated total reflection sampling technique. Thermogravimetric Analysis (TGA) was performed using Shimadzu TGA-60H thermo-balance. This apparatus operated within a temperature range of approximately 25 to 900 °C, with a synthetic air flow rate of 50 mL/min and a heating rate of 10 °C/min in an alumina crucible. Inductively coupled plasma mass spectrometry (ICP-MS) was conducted using a PerkinElmer ICP-OES Optima 8000. Elemental combustion analysis (EA) was carried out with a vario MICRO cube CHNOS elemental analyzer from Elementar Analysensysteme GmbH. Nitrogen adsorption-desorption isotherms and pore size distributions were measured at 77 K with a Quantachrome Quadrasorb SI instrument. Prior to each measurement, the samples were degassed at 150 °C under a vacuum of 0.5 Torr for 15 hours. The specific surface area of each material was calculated from the adsorption branch data (P/P0 < 0.3) using the Brunauer-Emmett-Teller (BET) method. The sample morphologies were examined using a scanning electron microscope (SEM) (Zeiss LEO 1550-Gemini) with an energy-dispersive X-ray (EDX) detector (Oxford Instruments X-MAX). Transmission electron microscopy (TEM) images were captured using a JEOL JEM F200 and a double Cs corrected JEOL JEM-ARM 200F operating at 80 kV, equipped with a cold-field emission gun and a high-angle silicon drift EDX detector (Jeol JED 2300, with a solid angle up to 0.98 steradians and a detection area of 100 mm²). The optical properties and charge carrier behaviors were analyzed using UV-vis diffuse reflectance spectroscopy (UV-vis DRS, UV-2600, Shimadzu, Japan), electron paramagnetic resonance (EPR, Bruker EMXnano), steady-state photoluminescence (PL) with a Jasco FP-8300 fluorescence spectrometer at an excitation wavelength of 365 nm and time-resolved PL (TRPL), recorded on fluorescence lifetime spectrometer (FluoTime 250, PicoQuant) equipped with PDL 800-D picosecond pulsed diode laser drive. The average lifetime (τ_ave_) is calculated as follows:

τ_ave_ = (A_1_ τ_1_^2^ + A_2_ τ_2_^2^+ A_3_ τ_3_^2^) / (A_1_ τ_1_ + A_2_ τ_2_ +A_3_ τ_3_) (1)

2. Electrochemical Measurements

Rotating ring-disk electrode (RRDE) or rotating disk electrode (RDE) technique was used to measure the number of transferred electrons (n) of the sample in the ORR reaction. The electrochemical measurement used a three-electrode system, wherein Ag/AgCl and Pt ring serve as reference and counter electrodes with O_2_-saturated 0.2 M Na_2_SO_4_ solution as electrolyte. The speed of RRDE was set different rotating speeds (from 400 to 1600 rpm) and the potential range was set to 0–1.0 V vs. RHE. To prepare the catalyst ink, 6 mg of the catalyst and 6 mg of black carbon was fully ground and dispersed in pure water (490 µL), isopropanol (490 µL), and Nafion solution (20 µL) via sonication for 1 h. Next, 11 µL of ink was dropped on the RRDE electrode and dried at room temperature (the catalyst loading was approximately 0.28 mg cm^-2^). For the RRDE testing, linear sweep voltammetry (LSV) is performed on the disk electrode, while a constant potential (e.g. 1.2 V vs. RHE) is applied on the ring electrode. When H_2_O_2_ is produced on the disk, it can diffuse to the ring then be detected and the number of transferred electrons (n) is often calculated according to the formula (2), where *I_r_* is the current at the ring electrode, *I_d_* is the current on the disk electrode, and N is the collection efficiency (*N* = 0.37). For the RDE measurements, the Koutecky–Levich (K–L) equation (3) is applied to calculate n, where *I* is the measured steady-state current derived from the ORR (mA cm^−2^), *I_K_* is the kinetic current of the reaction with active substances on the electrode surface, *F* is the Faraday constant (96458 C mol^−1^), *A* is the geometric area of the work electrode, *D_0_* is the diffusion coefficient of O_2_ in the electrolyte (cm^2^ s^−1^), *ω* is the angular rotation speed, *ν* is the kinematic viscosity of the electrolyte (cm^2^ s^−1^), $C_{O_{2}}$ is the saturated concentration of O_2_ in the solution (mol cm^−3^).

$n=\frac{I_{d}}{I_{d} + I_{r} /N} *100 \%$ (2)

$\frac{1}{I} = \frac{1}{I_{k}} + \frac{1}{0.620nFAD_{0}^{2/3} \omega^{1/2}v^{-1/6}C_{O_{2}}}$(3)

The selectivity of H_2_O_2_ (*S*) is calculated using the following equation (4):

$S=n*\frac{I_{r} / N}{I_{d} + I_{r} /N} *100 \%$ (4)

3. Photoelectrochemical measurements

All photoelectrochemical measurements were carried out in a three-electrode configuration, with a Pt wire and Ag/AgCl as counter and reference electrodes, respectively. The electrocatalysis experiments were conducted with a Gamry Interface 1010E potentiostat. To prepare the working electrode, F-doped Tin Oxide (FTO) glass (3 x 1 cm) substrates were cleaned sequentially with detergent, distilled H_2_O and ethanol for 15 min each to remove organic impurities. Half of the FTO area was protected with a Scotch tape. A catalyst ink was obtained by mixing 5 mg of photocatalyst powder, 0.5 mL of H_2_O and 20 μL of 5 wt.% Nafion by sonication for 30 min. Then, 50 μL of catalyst slurry was pipetted onto the FTO electrode and dried at 60 ℃ and further heated at 120 ℃ for 1 h to improve adhesion. All measured potentials were converted to reversible hydrogen electrode (RHE) according to the following equation (4):

*E_RHE_* = *E_Ag/AgCl_* + 0.059*pH + 0.197 (4)

3.1 Transient photocurrent response (TPR)

The photocurrent response was measured at 0 V versus reference electrode in 0.5 M aqueous Na_2_SO_4_ solution under white LED (100 mW cm^-2^) illumination using a Gamry Interface 1010E potentiostat.

3.2 Electrochemical impedance spectroscopy (EIS)

For *EIS*, the same electrodes were used as described above and the measurements were done in a frequency range of 10 kHz to 1 Hz. The data were fitted to a full semicircle using Z-View software.

3.3 Mott–Schottky measurements (MS)

*MS* measurements were performed in a Biologic MPG-2 system at different frequencies using the same electrodes as described above.

4. Radical quenching experiments

A sample vial was filled with 5 mg 2%*Ox*-KPHI powder in 2 mL water containing 10% glycerin along with a 10 mM sacrificial reagent: AgNO_3_ for e^-^, NaS_2_O_3_ for ·O_2_^-^, or t-butyl alcohol (TBA) for ·OH. The mixture was then purged with O_2_ gas for 1 min. The vial was irradiated under stirring using two 50W LED lamps (λ = 410 nm) for 1 h. The amount of generated H_2_O_2_ was quantified by spectrophotometry.

5. EPR experiment

The presence of ·O_2_⁻ in the reaction system was measured using spin trapping electron paramagnetic resonance (EPR). Typically, 5,5-Dimethyl-1-pyrroline N-oxide (DMPO) was employed as the spin-trapping reagent to detect radicals. The measurements were conducted in a H_2_O/MeOH mixture (1:4, 500 μL) containing 1 mg of the sample and 0.1 mmol DMPO, with a 410 nm LED as the light source.

6. Evaluation of photocatalytic performance

Initially, 5 mg of the catalyst was dispersed in 2 mL of an aqueous glycerin solution at a specified concentration, and O_2_ gas was bubbled through the mixture for 1 min. The reactor was then irradiated under stirring with two 50 W LED lamps (λ = 410 nm) for 1 h. After irradiation, the suspension was centrifuged at 10,000 rpm for 10 minutes to separate the catalysts from the solution. The amount of generated H_2_O_2_ was quantified spectrophotometrically using the titanium oxalate method. Specifically, a 10 g/L solution of K_2_[TiO(C_2_O_4_)_2_]·2H_2_O was prepared by dissolving the compound in 450 mL of water and 50 mL of H_2_SO_4_ to prevent complex precipitation. Then, 1.5 mL of this reagent was mixed with 0.5 mL of the supernatant from the photocatalytic experiment. The resulting solutions, diluted as necessary, were analyzed using a UV-vis spectrometer, measuring absorbance at 400 nm. A calibration curve was constructed with external H_2_O_2_ samples of known concentrations ranging from 0 to 10 mmol/L, exhibiting a linear analytical response (R² = 0.9999) .

7. Apparent quantum yield (AQY) estimation

The AQY was measured using different monochromatic LEDs (365, 410, 465, 525 and 620 nm). The AQY was obtained by the following equation (5):

$\mathrm{AQY}\left( \% \right)=\frac{2*Rproduct*N_{A}*hc}{I*A* \lambda}* 100$(5)

where *R_product_* is the production rate of H_2_O_2_ molecules (mol s^−1^) after the 1^st^ hour of photocatalytic reaction, *N_A_* is Avogadro constant (6.022 × 10^23^ mol^−1^), *h* is the Planck constant (6.626 × 10^−34^ J s^−1^) multiplied by *c* the speed of light (3 × 10^8^ m s^−1^) giving (1.98644586 × 10^−25^ J m), *A* is the irradiation area (cm^2^), *I* is the intensity of irradiation light (W cm^−2^), and *λ* is the wavelength of the monochromatic light (nm).

8. Femtosecond transient absorption spectroscopy (fs-TAS)

The femtosecond transient absorption spectroscopy (fs-TAS) measurements were made using pump-probe spectroscopy. The fundamental ultrashort laser pulses were generated at 800 nm from the Ti:Sapphire laser (Libra F, Coherent Inc.), of which 90 % was coupled to an optical parametric amplifier (OPA) from Topas C, Light Conversion Ltd to produce the pump beam (for 410 nm, 0.03 mJ/ cm^2^). The remaining 10 % was passed through a delay line for a transient response up to 5 ns and then a water cuvette to generate the probe beam in the visible range (400 – 770 nm). The transient absorption responses of the visible range probe beam were measured using an ExciPro TA spectrometer (CDP, Inc.), with a Si CCD diode array. The time resolution of the instrument was 100 fs.

The measured data was corrected against group velocity dispersion and fitted globally to estimate characteristic time constant and the spectra of intermediate states. Typically, an initial fit model was a sum of exponentials plus a constant function as shown as in equation (**6**) below:

$\Delta A \left( \lambda, t \right)=A_{0} \left( \lambda\right)+\sum_{i=1}^{N} A_{i} \left( \lambda\right){exp}^{\frac{-t}{\tau_{i}}} \ldots($6)

where ΔA (λ,t) was the transient absorption at a specific wavelength and time; A_0_ (λ) was the response independent of the delay time; A_i_ (λ) were the pre-exponential factors or decay associated spectra (DAS); and τ_i_ were the time constants for each decay component.

For the fs-TAS measurements, 5 mg of each of the *2%Ox*-KPHI and KPHI powder samples were prepared in 5 mL of milliQ water. The samples were sonicated for an hour in a bath sonicator and centrifuged at 2500 rpm for 10 minutes to achieve stable dispersions in water. These were used as stock solutions and diluted appropriately to make a total of six samples; including (a) a control sample of each in milliQ water alone, (b) a sample of each in 3.5 % glycerin and (c) a sample of each in 10 % glycerin. The glycerin acts as a sacrificial electron and proton donor. All measurements were made in quartz cuvettes of path length 2 mm under normal atmospheric conditions of oxygen, temperature, and pressure.

9. DFT calculation

Density functional theory (DFT) implemented via materials studio with the CASTEP was used to determine the most stable adsorption configurations of *O, *OOH, and *H_2_O_2_. The Perdew-Burke-Ernzerhofer (PBE) generalized gradient approximation (GGA) was used to describe the electronic exchange and correlation effects of exchange-correlation interaction. The core electrons were represented by the Vanderbilt-type ultrasoft pseudopotentials with Koelling–Harmon relativistic treatment. Energy cutoﬀ was set as 450 eV and k-point was 2*2 *1. The self-consistent ﬁeld (SCF) tolerance and Max. force was converged to < 1 × 10^−5^ eV and <0.03 eV Å^−1^, respectively. The Max. stress and Max. displacement were set as 0.05 GPa and 0.001 Å, respectively.

**Figure S1.** Optical images of KPHI and oxamide-modified KPHI powders.


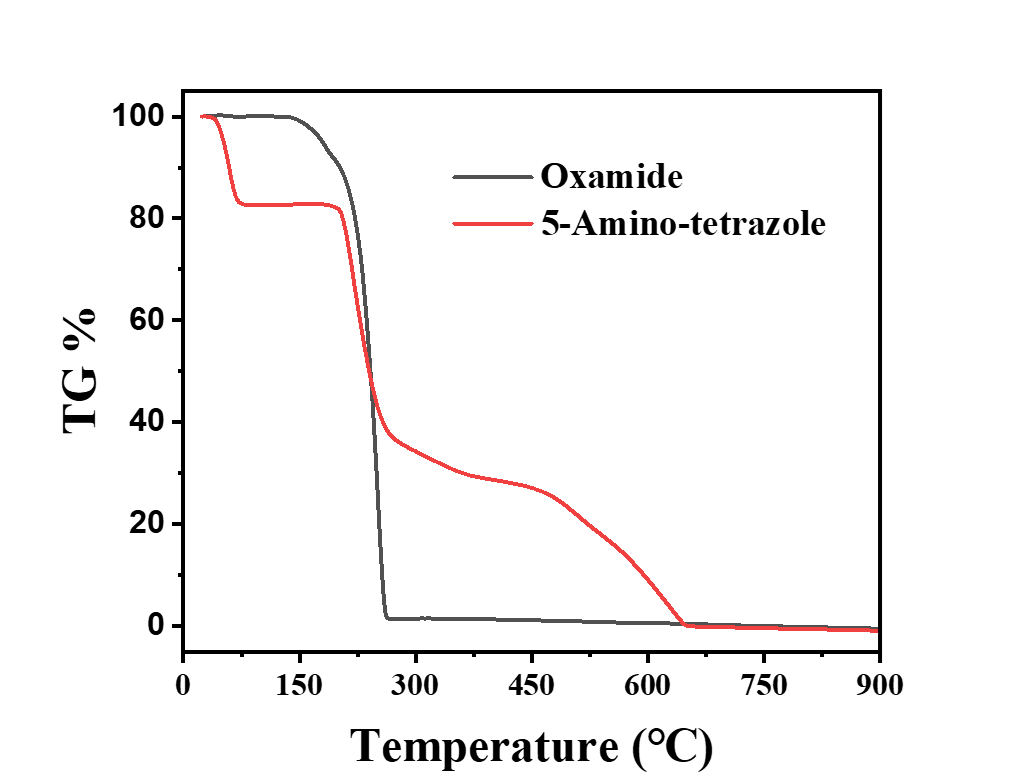


**Figure** **S2.** TG curves of 5-Amino-tetrazole and oxamide recorded under N_2_ atmosphere (10 K min^-1^).


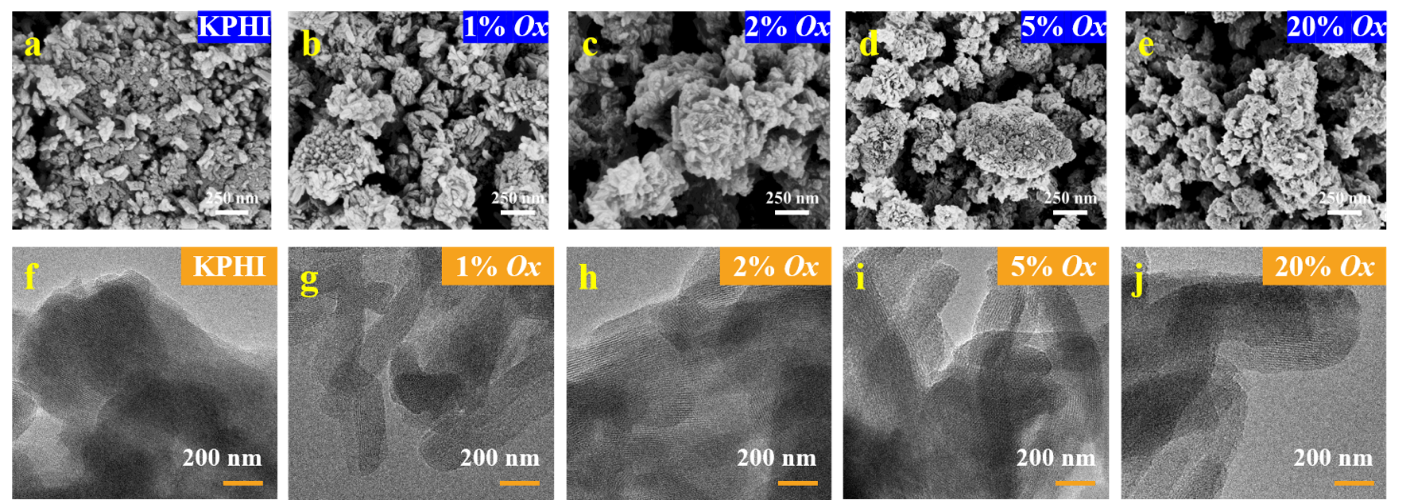


**Figure** **S3.** (a–e) SEM micrographs and (f–j) HR-TEM images of KPHI and *x*%*Ox*-KPHI samples.


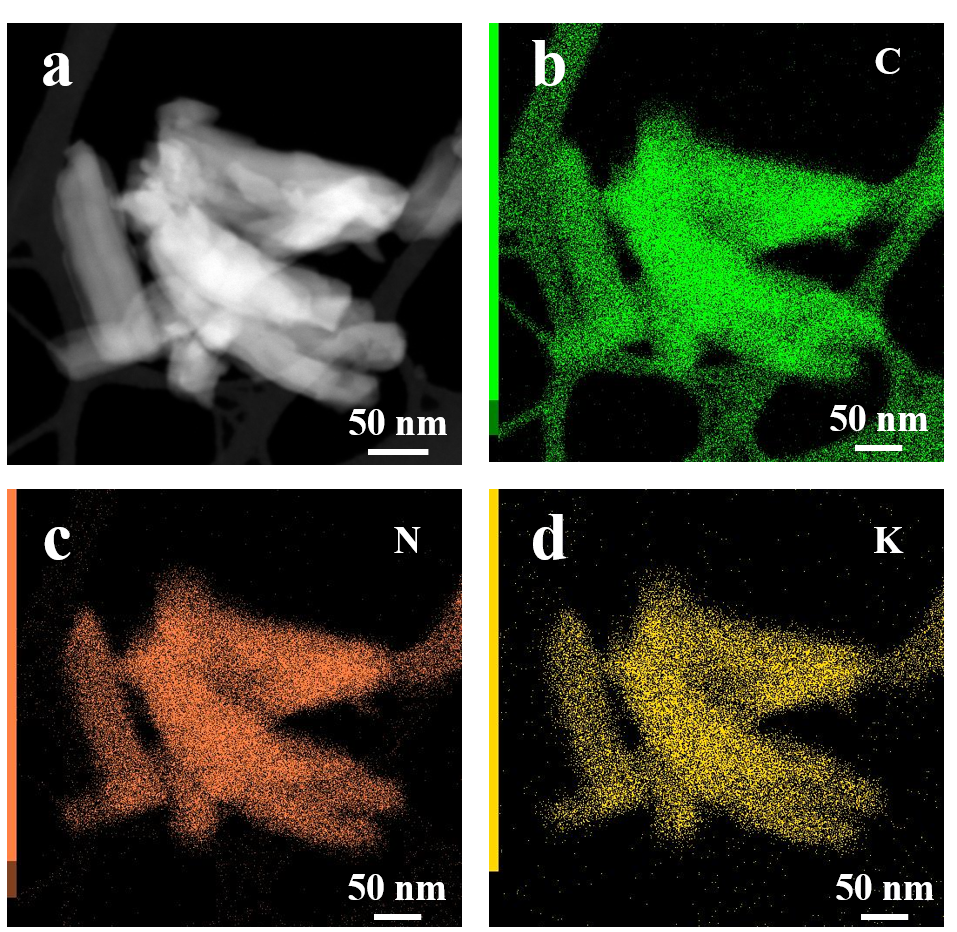


**Figure** **S4.** (a) HR-TEM image of *2%Ox*-KPHI. and (b–d) its corresponding elemental mapping images

**Table** **S1.** The relative ratios of C, N and O elementals in all samples determined by elemental analysis and the total amount of alkali metals in all catalysts via ICP.

| **Samples** | **C (%)** | **N (%)** | **H (%)** | **K (%)** | **Li (%)** |
| --- | --- | --- | --- | --- | --- |
| KPHI | 26.63 | 43.15 | 2.44 | 9.7 | 0.18 |
| 1% *Ox*-KPHI | 27.24 | 43.06 | 2.46 | 10.1 | 0.22 |
| 2% *Ox*-KPHI | 27.38 | 42.90 | 2.51 | 10.3 | 0.19 |
| 5% *Ox*-KPHI | 27.38 | 42.84 | 2.55 | 11.2 | 0.31 |
| 20% *Ox*-KPHI | 28.20 | 42.52 | 2.48 | 10.2 | 0.42 |


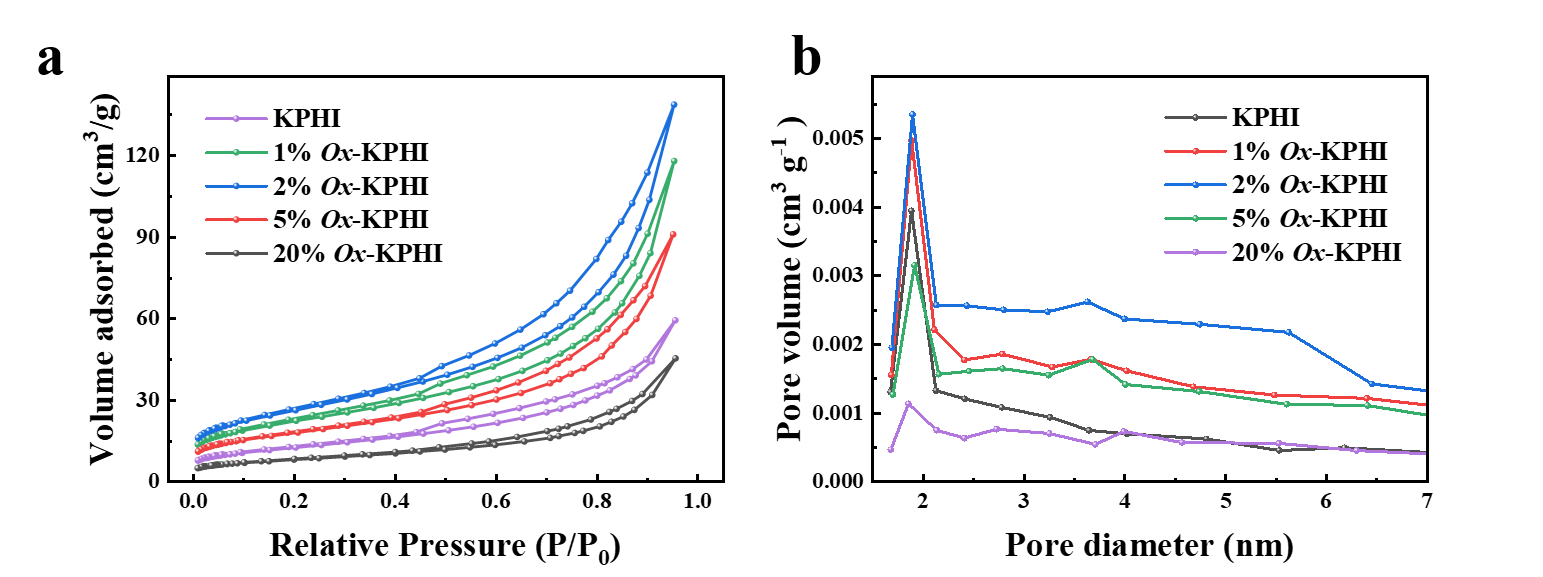


**Figure** **S5.** (a) N_2_ adsorption/desorption isotherm at 77K and (b) BJH pore size distribution from N2 adsorption branch at 77K of KPHI and *x*% *Ox*-KPHI.

**Table** **S2.** BET specific area pore volume and diameter size of all prepared samples.

| **Sample** | **S_BET_ m^2^ g^-1^** | **V_p_ cm^3^ g^-1^** | **D_p_ nm** |
| --- | --- | --- | --- |
| KPHI | 44.496 | 0.086 | 1.883 |
| 1% *Ox*-KPHI | 78.350 | 0.172 | 1.883 |
| 2% *Ox*-KPHI | 92.901 | 0.206 | 1.893 |
| 5% *Ox*-KPHI | 54.841 | 0.133 | 1.913 |
| 20% *Ox*-KPHI | 28.911 | 0.066 | 1.853 |


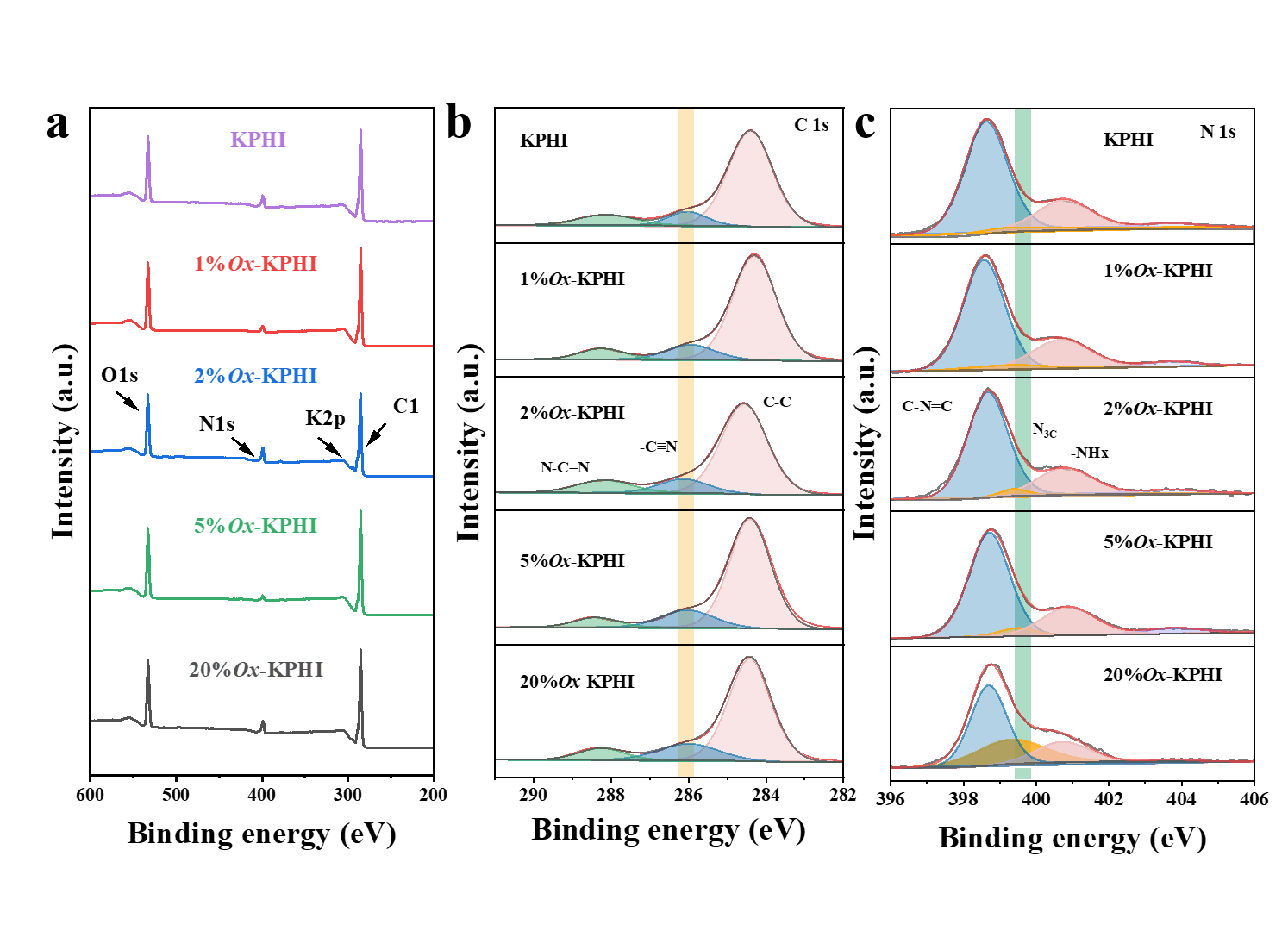


**Figure** **S6.** (a) XPS full spectra, (b) high-resolution C 1s and (c) N 1s of KPHI and *x*% *Ox*-KPHI.

**Table** **S3.** XPS N atom ratio.

| **Samples** | **N_2C_** | **N_3C_** | **N_2C_/N_3C_** |
| --- | --- | --- | --- |
| KPHI | 0.72 | 0.22 | 3.27 |
| 1% *Ox*-KPHI | 0.67 | 0.23 | 3.19 |
| 2% *Ox*-KPHI | 0.66 | 0.25 | 2.68 |
| 5% *Ox*-KPHI | 0.64 | 0.26 | 2.67 |
| 20% *Ox*-KPHI | 0.55 | 0.27 | 2.04 |

**
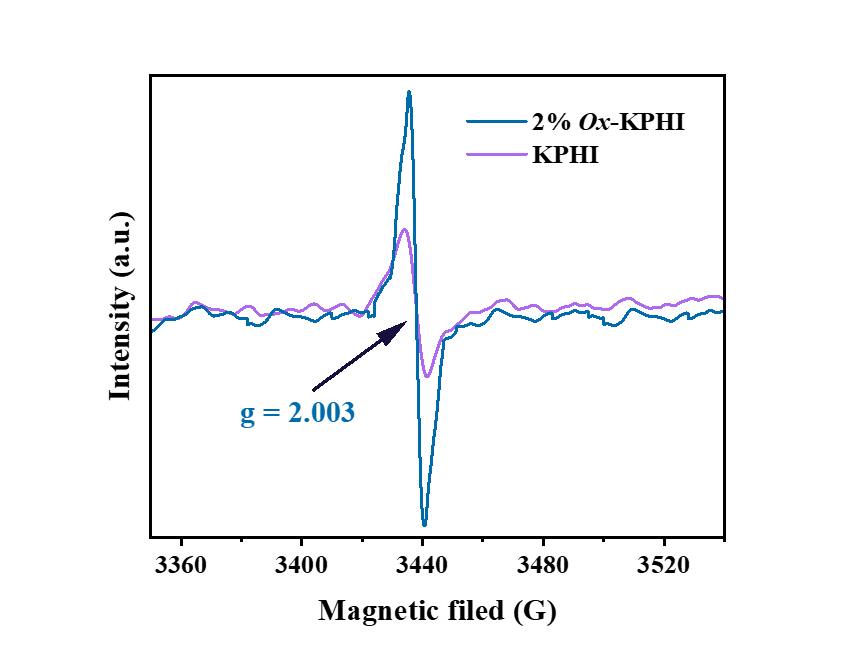
**

**Figure** **S7.** EPR signals of KPHI and 2% *Ox*-KPHI.

**
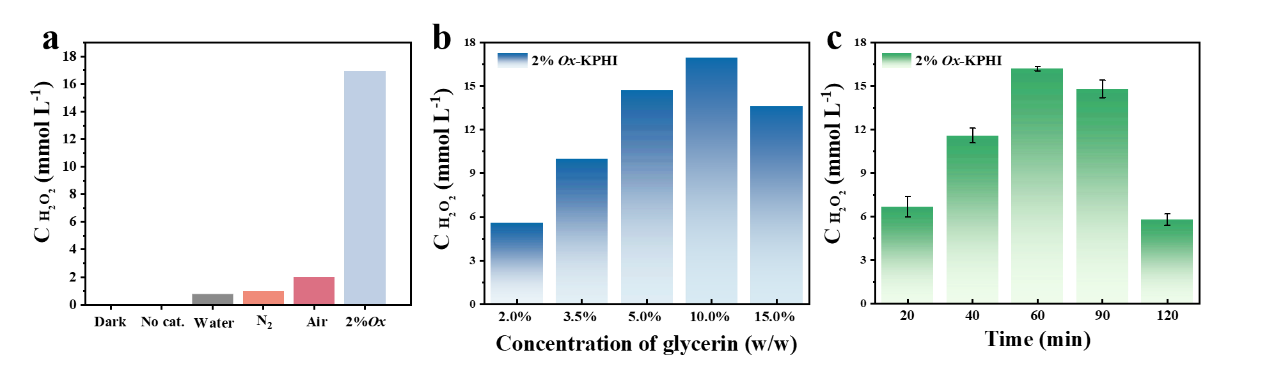
**

**Figure** **S8.** (a) Photocatalytic H_2_O_2_ production under different testing conditions, (b) different w/w concentration of glycerin and (c) different reaction time of 2% *Ox*-KPHI.


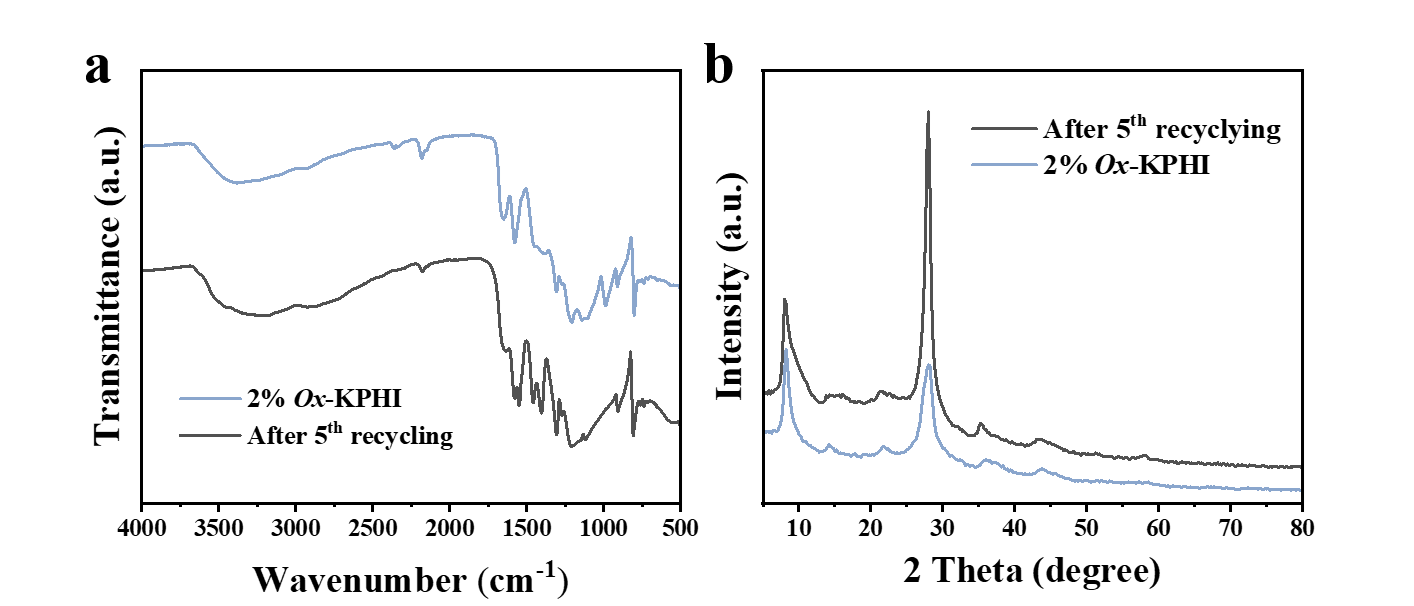


**Figure** **S9.** (a) FTIR spectra and (b) XRD patterns of 2% *Ox*-KPHI before and after H_2_O_2_ production recyclability tests.

**Table** **S4.** Comparison of H_2_O_2_ production yields and AQY values with other carbon nitride photocatalysts.

| **Photocatalyst** | **Modification** | | **Synthesis method** | | **Conditions** | **H_2_O_2_ yields**  **(μmol·g^-1^·h^-1^)** | **AQY (%)** | **Ref.** |
| --- | --- | --- | --- | --- | --- | --- | --- | --- |
| **Bulk g-C_3_N_4_** | — | Calcination | | Xe-lamp (λ>420nm) | | 125 | **7%**  **420nm** | **1** |
| **Nv-g-C_3_N_4_ nanosheets** | N vacancy | Thermal condensation | | Xe-lamp (λ>420nm) | | 1768 | **10.5%**  **420nm** | **2** |
| **Sb-doped**  **g-C_3_N_4_** | Sb doping | Thermal polymerization | | LED_420nm_ | | 3480 | **17.6%**  **420 nm** | **3** |
| **WSe_2_/g-C_3_N_4_** | Z-scheme heterojunction | Calcination Solvothermal | | Xe-lamp  (λ > 420nm) | | 40.62 | **7.18%**  **420 nm** | **4** |
| **Au/g-C_3_N_4_** | Au Co-catalyst | Carbon-layered stabilized | | Xe-lamp  (λ > 420nm) | | 82.5 | **3.63%**  **400 nm** | **5** |
| **g-C_3_N_4_ with hole defects** | Hole defects | Photo-assisted heating | | AM1.5  (λ > 420 nm) | | 5.81 | **10.2%**  **420 nm** | **6** |
| **HTCN** | H/T junction | Thermal condensation | | visible light (400 < λ < 800 nm) | | 22825.5 | **21.5%**  **420 nm** | **7** |
| **PDI/CNA** | D-A structure | Thermal condensation | | LED  (400 nm ≤ λ ≤ 760 nm ) | | 1605.32 | **27.18%**  **400 nm** | **8** |
| **Alkali metal-**  **doped Nv-g-C_3_N_4_** | N vacancy,  K, Na doping | Thermal copolymerization | | 300 W Xe-lamp  (λ > 400 nm) | | 3080 | **6.8%**  **400 nm** | **9** |
| **Nv–CN–CN** | N vacancy,  –C≡N group | Two-step calcination | | 300 W Xe-lamp  (λ ≥ 420 nm) | | 3093 | **22.1%**  **420 nm** | **10** |
| **KPHI** | — | Calcination | | LED_410nm_ | | **5876** | **32.08%**  **410 nm** | **this work** |
| ***Ox*-KPHI** | Oxamide induced | Calcination | | LED_410nm_ | | **6772** | **40.72%**  **410 nm** | **this work** |


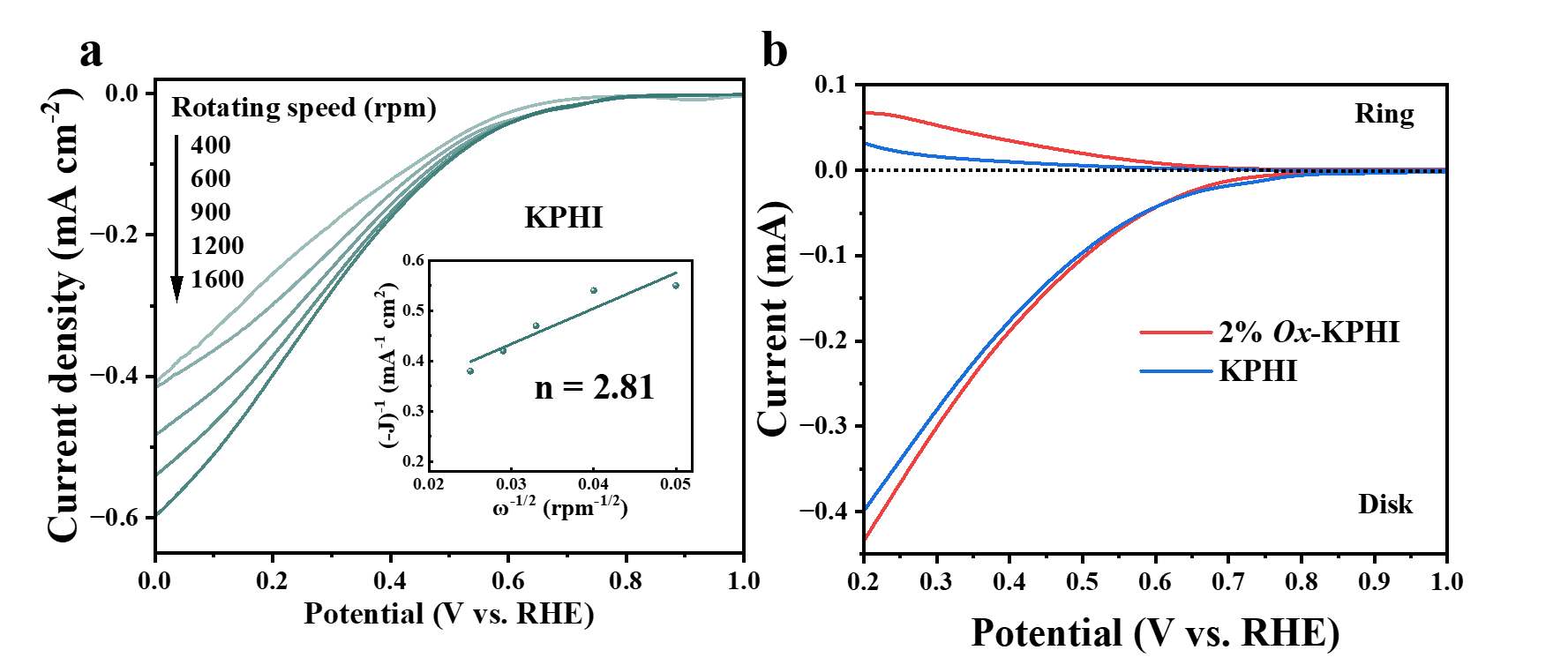


**Figure** **S10.** (a) Linear sweep voltammetry (LSV) curves of KPHI recorded on a rotating disk glassy carbon electrode with inset graph of K-L and (b) RRDE polarization curves over KPHI and 2% *Ox*-KPHI at 1600 rpm in O_2_-saturated 0.2 M Na_2_SO_4_ with ring current (upper part) and disk current (bottom part).

**
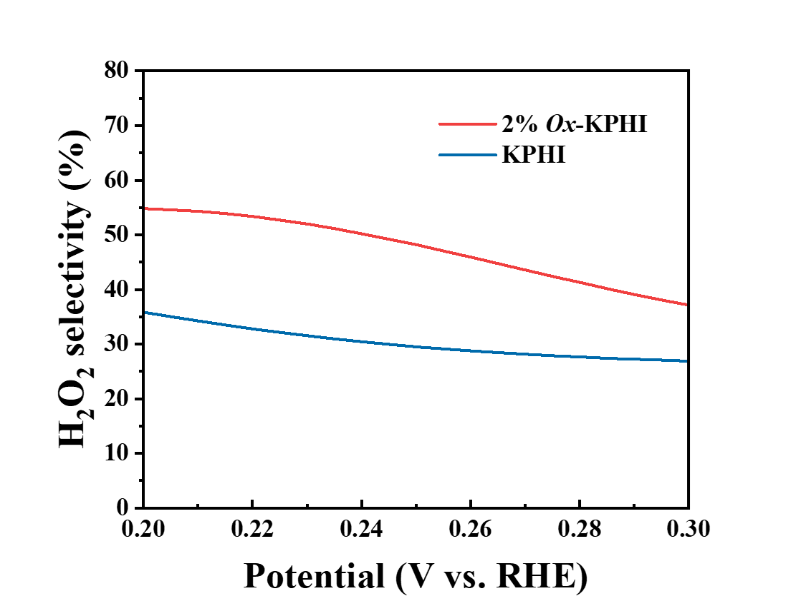
**Figure S11. H_2_O_2_ selectivity as a function of the applied potential.

**
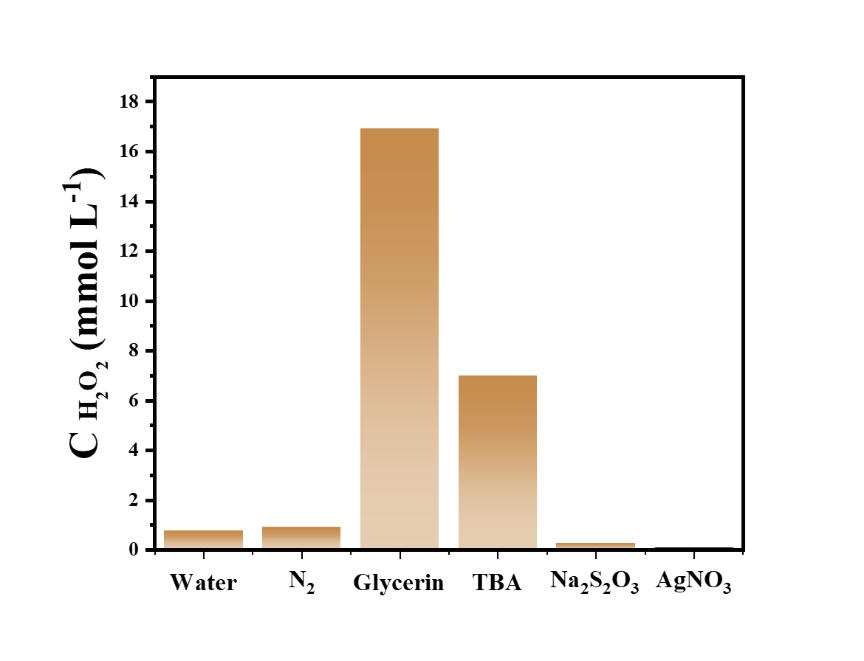
**

**Figure** **S12.** Influence of different scavengers on the photocatalytic H_2_O_2_ production of *2%Ox*-KPHI.

**
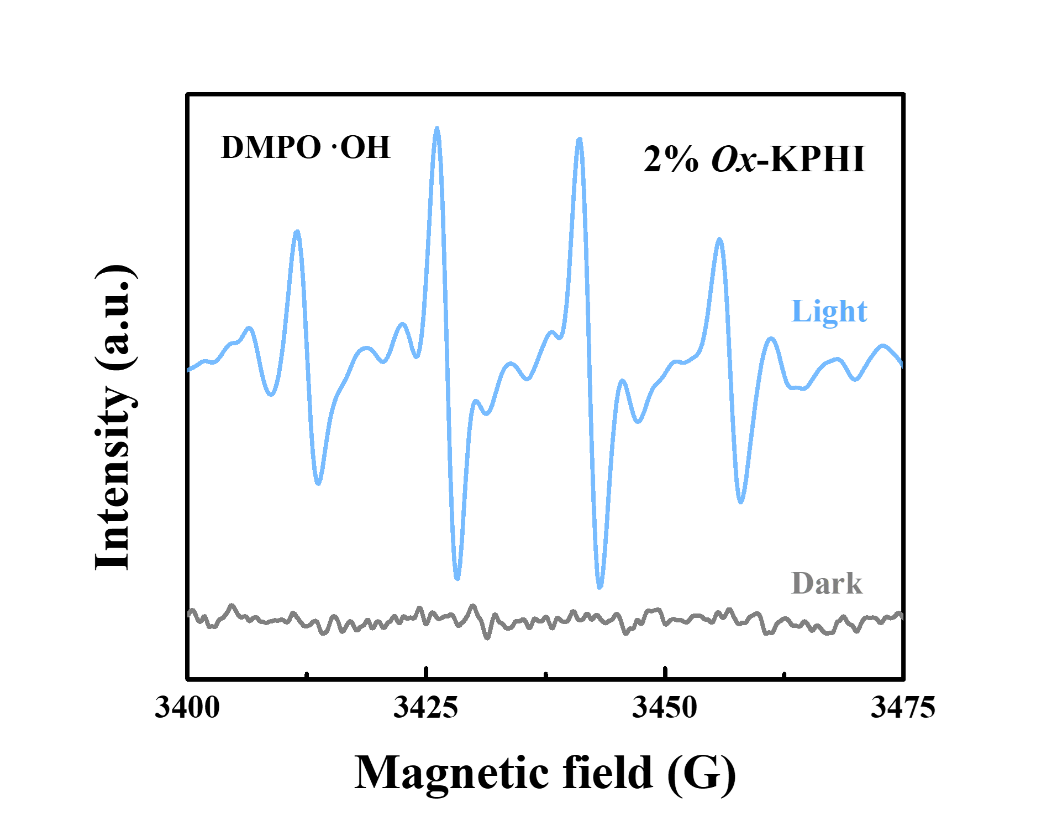
Figure S13.** EPR spectra of DMPO-⋅OH over *2%Ox*-KPHI


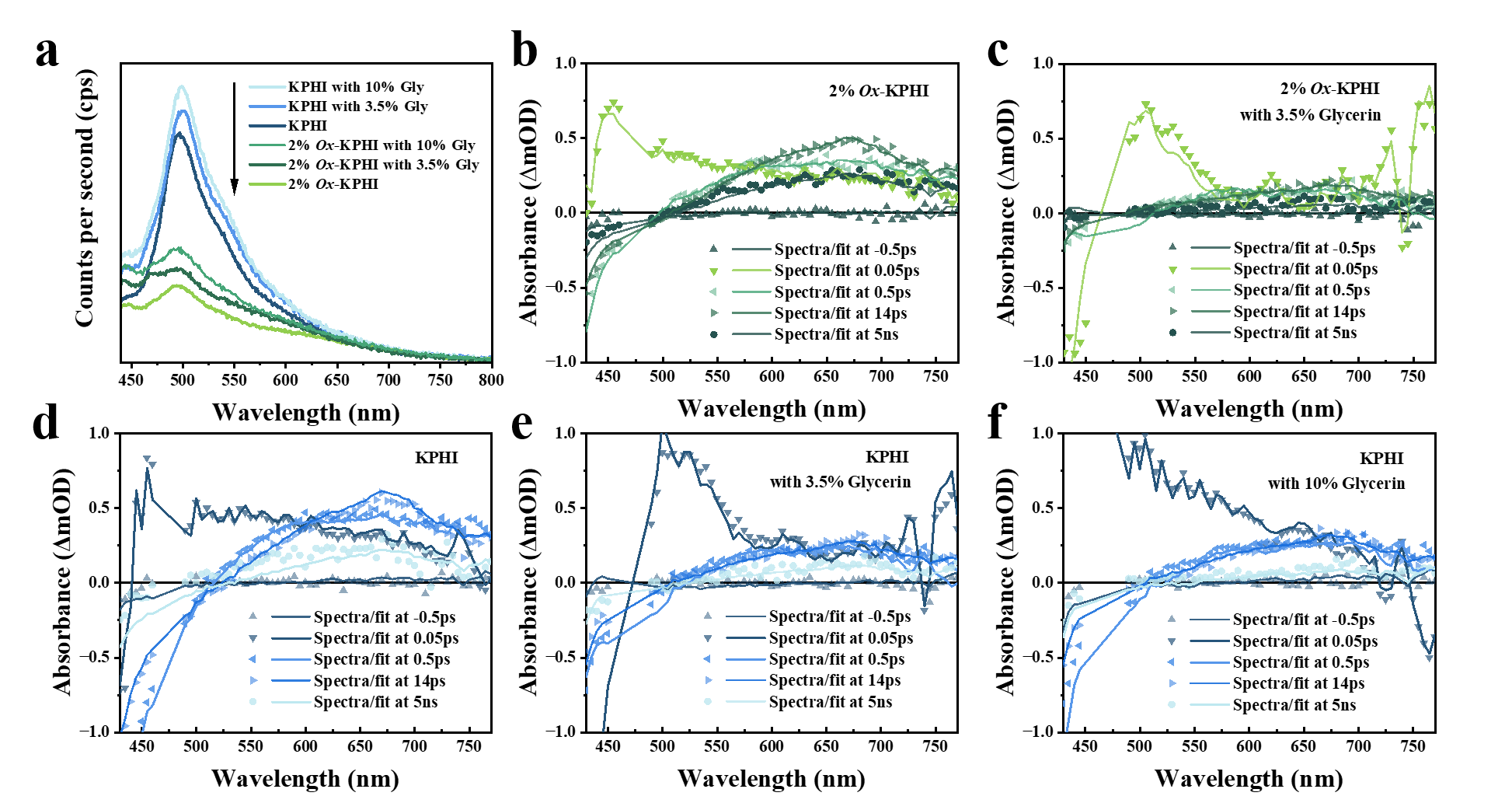


**Figure** **S14.** (a) The steady state emission spectra of KPHI and *2%Ox-*KPHI normalized to the absorbance at 404 nm in different concentrations of glycerin. Femtosecond transient absorption spectroscopy (fs-TAS) differential spectra at different delay times for (b) KPHI in water (c) KPHI in 3.5 % glycerin (d) KPHI in 10 % glycerin (e) *2%Ox*-KPHI in water (f) *2%Ox*-KPHI in 10 % Glycerin. (410 nm excitation, 0.03 mJ/ cm^2^).


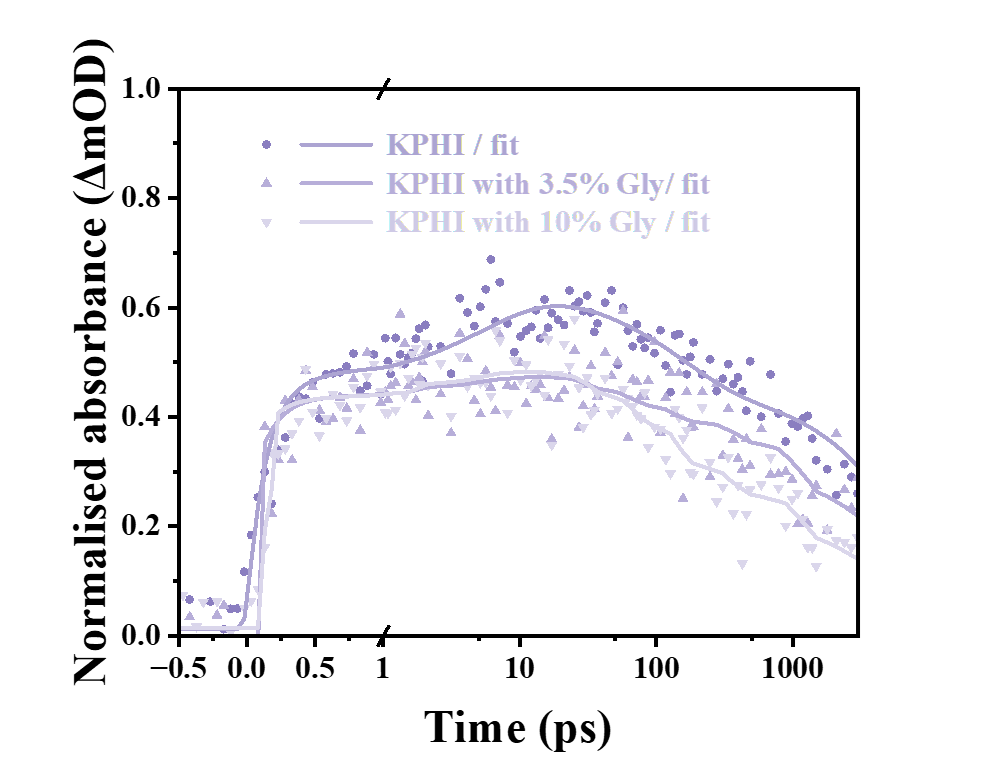


**Figure** **S15.** The fs-TAS decays at 675 nm monitoring wavelength for KPHI in different concentrations of Glycerin. (410 nm excitation, 0.03 mJ/ cm^2^)

**Table** **S5.** Results of multiexponential fitting for the fs-TAS decays.

| **Samples** | **Glycerin** | **τ1 (ps)** | **τ2 (ps)** | **τ3 (ps)** | **τ4 (ns)** |
| --- | --- | --- | --- | --- | --- |
| **KPHI** | - | 0.1 | 5.9 | 147.3 | 7.8 |
| **KPHI** | 3.5 % | 0.1 | 12.9 | 99.1 | 9.0 |
| **KPHI** | 10 % | 0.1 | 9.7 | 166.5 | 5.6 |
| **2% *Ox*-KPHI** | - | 0.1 | 3.5 | 161.1 | 12.4 |
| **2% *Ox*-KPHI** | 3.5 % | 0.3 | 8.1 | 688.5 | 6.5 |
| **20% *Ox*-KPHI** | 10 % | 0.1 | 8.4 | 690.2 | - |

**Figure S16.** DFT simulation of (a) KPHI and (b) *Ox*-KPHI structure, which is shown to have a distorted structure.

References

[1] Y. Shiraishi, S. Kanazawa, Y. Sugano, D. Tsukamoto, H. Sakamoto, S. Ichikawa, T. Hirai, ACS Catal. **2014**, 4, 774–780.

[2] H. Zhao, C. Shi, Q. Li, X. Wang, G. Zeng, S. Ye, B. Jiang, J. Liu, Mater. Today Energy **2022**, 24, 100926.

[3] Z. Teng, Q. Zhang, H. Yang, K. Kato, W. Yang, Y.-R. Lu, S. Liu, C. Wang, A. Yamakata, C. Su, B. Liu, T. Ohno, *Nat. Catal.* **2021**, 4, 374–384.

[4] W. Wang, W. Gu, G. Li, H. Xie, P. K. Wong, T. An, Environ. Sci.: Nano **2020**, 7, 3877–3887.

5] X. Chang, J. Yang, D. Han, B. Zhang, X. Xiang, J. He, *Catalysts* **2018**, 8, 147

[6] L. Shi, L. Yang, W. Zhou, Y. Liu, L. Yin, X. Hai, H. Song, J. Ye, Adv. Energy Mater. **2018**, 14, 1703142.

[7] Y. Zhang, Q. Cao, A. Meng, X. Wu, Y. Xiao, C. Su, Q. Zhang, Adv. Mater. **2023**, 35(48), 2306831.

[8] J. Hu, C. Chen, H. Yang, F. Yang, J. Qu, X. Yang, W. Sun, L. Dai, C. M. Li, Appl. Catal. B **2022**, 317, 121723.

[9] Q. Bai, Y. Huang, Z. Chen, Y. Pan, X. Zhang, Q. Long, Q. Yang, T. Wu, T-Z. Xie, M. Wang, H. Luo, C. Hu, P. Wang, Z. Zhang, Nano Res. **2023**, 16, 4524–4530.

[10] X. Zhang, P. Ma, C. Wang, L. Gan, X. Chen, P. Zhang, Y. Wang, H. Li, L. Wang, X. Zhou, K. Zheng, Energy Environ. Sci. **2022**, 15, 830–842.
